# Herbal Active Ingredients: Potential for the Prevention and Treatment of Acute Lung Injury

**DOI:** 10.1155/2021/5543185

**Published:** 2021-06-22

**Authors:** Wen-Ying Yu, Chun-Xiao Gao, Huan-Huan Zhang, Yue-Guo Wu, Chen-Huan Yu

**Affiliations:** ^1^College of Pharmacy, Hangzhou Medical College, Hangzhou, China; ^2^Key Laboratory of Experimental Animal and Safety Evaluation of Zhejiang Province, Hangzhou Medical College, Hangzhou, China; ^3^Institute of Cancer and Basic Medicine, Chinese Academy of Sciences, Hangzhou, China

## Abstract

Acute lung injury (ALI) is a life-threatening clinical syndrome with high morbidity and mortality. The main pathological features of ALI are increased alveolar-capillary membrane permeability, edema, uncontrolled migration of neutrophils to the lungs, and diffuse alveolar damage, resulting in acute hypoxemic respiratory failure. Glucocorticoids, aspirin, and other anti-inflammatory drugs are commonly used to treat ALI. Respiratory supports, such as a ventilator, are used to alleviate hypoxemia. Many treatment methods are available, but they cannot significantly ameliorate the quality of life of patients with ALI and reduce mortality rates. Herbal active ingredients, such as flavonoids, terpenoids, saponins, alkaloids, and quinonoids, exhibit advantages for ALI prevention and treatment, but the underlying mechanism needs further study. This paper summarizes the role of herbal active ingredients in anti-ALI therapy and progresses in the understanding of their mechanisms. The work also provides some references and insights for the discovery and development of novel drugs for ALI prevention and treatment.

## 1. Introduction

Acute lung injury (ALI) is a common clinical syndrome with high morbidity and mortality and is caused by various pathological and structural changes due to direct and indirect injury factors in lung tissues. The pathological feature of this disease is damage to alveolar epithelial and microvascular endothelial cells. The former can cause edema in the alveolar cavity, exudation of fibrin and collagen, and aggregation of neutrophils, eventually leading to lung consolidation. The latter increases vascular permeability, which causes the aggregation of inflammatory cells and leads to pulmonary vascular congestion and pulmonary interstitial edema [1]. Therefore, the main factor in ALI development is the imbalance in inflammatory response, which damages diffuse alveolar and pulmonary vascular endothelial cells and may lead to oxidation/reduction imbalance [2].

The international treatment for ALI mainly includes reduction in inflammation damage and suppression of respiratory failure. Anti-inflammatory drugs, such as glucocorticoids and aspirin, are commonly used in clinics. Respiratory support, such as ventilators, is employed to relieve hypoxemia. Although many treatments are available, the patient's quality of life remains poor, and mortality is not reduced [3]. The activities of active ingredients from herbs in preventing and treating ALI have been recently explored. These substances show a broad application prospect. However, despite their widespread existence and recognized biological activities, no integral review of their mechanisms of action for ALI has been undertaken. This review is aimed at integrating and discussing the most promising anti-ALI compounds isolated from herbs over the last 10 years (2011-2020). For the collection of relevant information, reviews and single publications were searched on PubMed, SciFinder, ScienceDirect, and similar databases with relevant keywords, such as flavonoids, terpenoids, saponins, alkaloids, quinonoids, phenols, organic acids, coumarins, lignans, and ALI. Only in vivo research articles have been selected, and the mechanisms of action have been discussed. This work provides important clues and direction for the discovery of anti-ALI drugs.

## 2. Potential Effects of Herbal Active Ingredients on Experimental ALI

### 2.1. Flavonoids

Flavonoids constitute a group of compounds with 2-phenyl chromone structures; they are widely present in natural plants and have a variety of biological activities, such as antioxidant, anti-inflammatory, anticancer, antibacterial, and antiviral activities [4]. Their anti-inflammatory and antioxidant activities render them useful preventive and therapeutic candidates for inflammatory lung diseases.

Naringin is a natural 2,3-dihydro-flavonoid derived from *Exocarpium Citri Grandis*. Chen et al. [5] found that naringin can protect paraquat- (PQ-) induced ALI by downregulating the levels of transforming growth factor-*β*1 (TGF-*β*1), tumor necrosis factor-*α* (TNF-*α*), tissue inhibitor of metalloproteinase-1 (TIMP-1), and matrix metalloprotein-9 (MMP-9) and increasing the activities of glutathione peroxidase (GSH-Px), superoxide dismutase (SOD), and heme oxygenase-1 (HO-1). The compound exhibits mucoactive effects in lipopolysaccharide- (LPS-) induced ALI in mice and beagle dogs by reducing goblet cell hyperplasia and excessive mucus secretion and promoting sputum excretion [6].

Tectorigenin, an isoflavone aglycone, is obtained from *Belamcanda chinensis*. Tectorigenin treatment can attenuate inflammatory cell number in bronchoalveolar lavage fluids (BALF), reduce the mRNA and protein expression levels of nuclear factor kappa b (NF-*κ*B) p65 in the lungs, improve SOD activity, and inhibit myeloperoxidase (MPO) activity. It exhibits a protective effect on LPS-mediated ALI in mice [7].

Baicalin is a flavonoid glycoside isolated from *Scutellaria baicalensis* and can alleviate ALI induced by various factors. Meng et al. [8] found that it can provide protection against LPS-induced severe lung injury in mice by activating the nuclear factor E2-related factor 2- (Nrf2-) mediated HO-1 signaling pathway, which suppresses inflammation and oxidative stress. Baicalin can ameliorate severe burn-induced remote ALI in rats by suppressing the Nod-like receptor pyrin domain-containing protein 3 (NLRP3) signaling pathway [9] and avian pathogenic *Escherichia coli-* (*E. coli-*) induced ALI in chicken by restraining the activation of the NF-*κ*B pathway [10].

Hesperidin is a flavanone glycoside, which is found mainly in citrus fruit peel. In sepsis-induced ALI models, treatment with hesperidin can provide protection against lung injury by attenuating the levels of B-cell lymphoma-2 (Bcl-2), caspase-3, heat shock protein 70 (Hsp70), toll-like receptor 4 (TLR4), and myeloid differential protein-88 (MyD88) in the lung tissues [11]. In an H1N1-induced ALI model, hesperidin exhibits efficacy in alleviating pulmonary inflammation and impairment by suppressing cytokine production through mitogen-activated protein kinase (MAPK) pathways in pulmonary microvascular endothelial cells [12]. In an LPS-induced ALI model, hesperidin pretreatment can modulate lung injury by inhibiting the infiltration of macrophages and suppressing the release of high-mobility group box-1 protein (HMGB1) [13].

**Figure 1:**
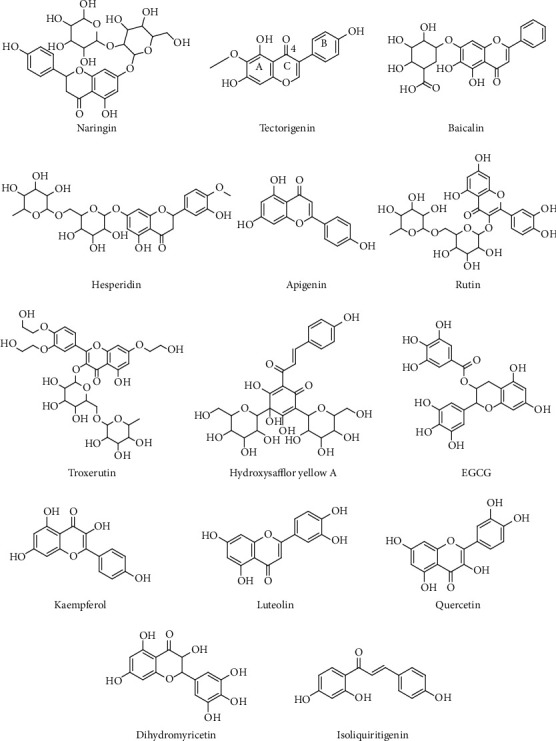
Chemical structures of representative flavonoids.

Apigenin (4′,5,7-trihydroxyflavone) is a natural flavonoid compound and abundantly present in common fruits and vegetables, such as celery, apple, and onion. It can reverse ALI and immunotoxicity in PQ-induced mice by increasing GSH-Px, catalase (CAT), and SOD activities and decreasing the levels of proinflammatory cytokines and malondialdehyde (MDA) [14]. Apigenin pretreatment can protect LPS-induced ALI in mice, and the protective mechanism of apigenin might be partially due to the inhibited activation of cyclo-oxygen-ase-2 (COX-2) and NF-*κ*B and subsequent decreased production of proinflammatory mediators [15].  Table 1 lists the potential effects of flavonoids on ALI. The structure of representative flavonoids is shown in Figure 1. In general, the key structural features of active flavonoids are unsaturation in the C ring (*Δ*^2^), the number and position of OH groups at the A and B rings, the carbonyl group at C-4 at ring C, and frequently, nonglycosylation of the molecules [16]. When these features are considered, one may infer that natural products, such as flavonoids, are useful in developing novel substances for ALI treatment.

### 2.2. Terpenoids

Terpenoids are compounds with the general formula of (C_5_H_8_)n, and their oxygenated derivatives and derivatives with different degrees of saturation can be considered natural compounds linked by isoprene or isopentane in various ways. Some terpenoids have antibacterial, antioxidant, tumor suppression, hepatoprotective, anti-inflammatory, and other biological activities [17, 18].

Andrographolide is a diterpenoid lactone extracted from *Andrographis paniculata* and commonly used to treat respiratory infection. Peng et al. [19] found that andrographolide can ameliorate ovalbumin- (OVA-) induced ALI in mice by inhibiting the reactive oxygen species- (ROS-) mediated activation of the NF-*κ*B and NLRP3 inflammasome signaling pathway. In addition, andrographolide contributes to the amelioration of lung inflammation and fibrosis induced by radiation and can suppress absent in melanoma 2 (AIM2) from translocating to the nucleus to sense DNA damage caused by chemotherapeutic agents or radiation in bone marrow-derived macrophages [20]. It exhibits antioxidative properties and can provide protection against cigarette smoke- (CS-) induced ALI by increasing Nrf2 activity [21].

Oridonin, isolated from *Rabdosia rubescens*, is an ent-kaurene diterpenoid that possesses antioxidative and anti-inflammatory effects. It can provide protection against LPS-mediated ALI by regulating Nrf2-dependent oxidative stress and Nrf2-independent NF-*κ*B and NLRP3 pathways [22] and alleviate hyperoxia-induced lung injury. Treatment with 10 mg/kg oridonin can attenuate lung pathology, reduce lung edema, decrease TNF-*α* and MDA levels, and increase interleukin- (IL-) 10 and GSH levels in the lungs of model mice [23].

Betulin and betulinic acid are natural pentacyclic triterpenoids derived from various plants, including white birch bark. Zhao et al. [24] found that betulin can reduce lung damage caused by cecal ligation and puncture (CLP) and improve the survival rates of rats. This protective effect may be mediated by its anti-inflammatory effect and NF-*κ*B and MAPK inhibition. Betulinic acid pretreatment can decrease the levels of oxidants and increase the levels of antioxidants in plasma and lungs, thereby reducing oxidative lung damage in CLP mice [25].

**Figure 2:**
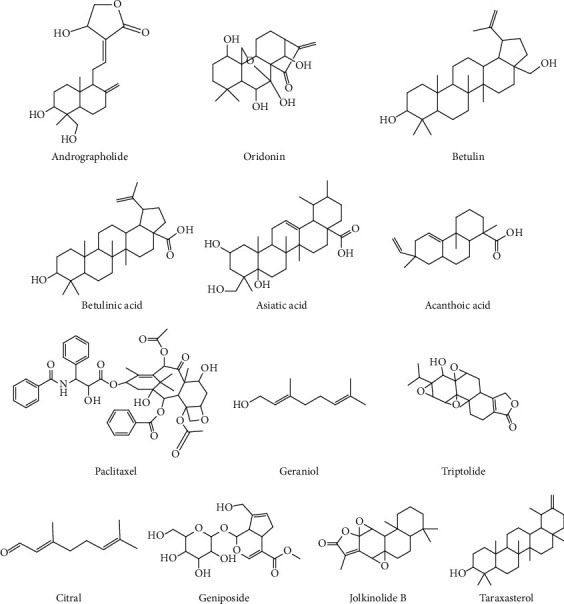
Chemical structures of representative terpenoids.

Asiatic acid is a pentacyclic triterpene derived from *Centellae asiaticae herba*. It can ameliorate LPS-induced histopathological changes in the lungs of mice by suppressing the production of inflammatory cytokines, and process is mediated by the blocked activation of TLR4-mediated NF-*κ*B signaling [26]. Asiatic acid exerts a considerable protective effect on spinal cord injury- (SCI-) induced ALI by blocking NLRP3 inflammasome activation and oxidative stress and by upregulating Nrf2 expression [26]. The potential effects of terpenoids from herbs on ALI are shown in Table 2. The structures of representative terpenoids are shown in Figure 2. Structure-activity analysis shows that the introduction of acyl groups into the mother nuclei of terpenoids improves the anti-inflammatory activity of terpenoids, and increasing the amount of acyl groups and lengths of acyl carbon chains enhances this effect. The type of substituents on the C rings of polycyclic terpenoids is the main reason for their anti-inflammatory activity, and rosin/pimonane diterpenoids and ingenane diterpenoids have good inhibitory activities against NO production [27].

### 2.3. Saponins

Saponins are glycosides composed of triterpene or spiral sterane and mainly distributed in terrestrial higher plants. Saponins have multiple biological activities, such as anti-inflammatory, antibacterial, and antiviral activities, and may prevent or treat ALI [28].

Asiaticoside, a triterpenoid saponin derived from *Centella asiatica*, possesses potential anti-inflammatory and antioxidant activities. Qiu et al. [29] found that asiaticoside can attenuate LPS-induced ALI in a dose-dependent manner by inhibiting NF-*κ*B p65 subunit phosphorylation and I*κ*B*α* degradation. Asiaticoside can provide protection against CLP-induced ALI, and its underlying mechanisms may be related to the activation of peroxisome proliferator-activated receptor-*γ* (PPAR-*γ*) to some extent, which suppresses MAPKs and the NF-*κ*B signaling pathway [30].

Platycodin D is the main triterpene saponin extracted from the Chinese herb *Platycodonis radix* and has anti-inflammatory and immunomodulatory activities. The anti-inflammatory effects of platycodin D may be associated with the activation of the liver x receptor *α* (LXR*α*)/ATP-binding cassette transporter A1 (ABCA1) signaling pathway, which disrupts the formation of lipid rafts by depleting cholesterol and preventing the translocation of TLR4 to lipid rafts, thereby blocking LPS-induced ALI [31]. Tao et al. [32] found that platycodin D pretreatment or posttreatment can improve ALI induced by LPS or BLM. Platycodin D can suppress apoptosis and inflammation by downregulating the levels of caspase-3, Bcl2-associated X protein (Bax), and NF-*κ*B and upregulating the level of Bcl-2 in lung tissues and improve the SOD activity in BALF.

Dioscin, a natural steroid saponin mainly derived from *Dioscorea villosa*, can serve as an anti-inflammatory agent. Treatment with dioscin can attenuate oxidative stress, lung inflammatory response, and ALI in BLM-challenged mice [31]. Furthermore, dioscin can also alleviate LPS-induced ALI by inhibiting TLR4 signaling pathways [33].

Ginsenosides form a class of steroid glycosides and triterpene saponins. As the main active ingredients in *Ginseng radix*, they have various pharmacological effects, such as immunity enhancement and antiageing and antitumor effects [34–36]. Ginsenosides include Rb1, Rg1, Rg3, GRh2, and other monomeric saponins. The ginsenoside Rb1 can provide protection against *Staphylococcus aureus-* (*S. aureus-*) induced ALI in mice. It can inhibit TNF-*α*, IL-1*β*, and IL-6 production and TLR2 activation. Furthermore, Rb1 effectively downregulates the phosphorylation of extracellular regulated kinases (ERK), p65, and c-Jun N-terminal kinase (JNK). These results demonstrated that Rb1 can alleviate lung injury by inhibiting TLR2-mediated NF-*κ*B and MAPK pathways [37]. Rg1 can relieve sepsis-induced ALI by upregulating SIRT1 to relieve endoplasmic reticulum (ER) stress and inflammation [38], and ginsenoside Rg1 pretreatment can attenuate ALI induced by hind limb ischemia-reperfusion (IR) by inhibiting the NF-*κ*B/COX-2 signaling pathway [39]. Rg3 treatment can mitigate LPS-induced pathological damage in the lungs by increasing the production of anti-inflammatory mediators and reducing the levels of proinflammatory cytokines. This process is mediated by MerTK-dependent activation of its downstream PI3K/AKT/mTOR pathway. These findings identified a new site of the specific anti-inflammatory mechanism of ginsenoside Rg3 [40]. Rg3 exhibits a protective effect against omethoate-induced ALI in rats, and the mechanisms may be related to its antioxidant potential and anti-inflammatory effects [41]. The anti-inflammatory effect of Rh2 has made it one of the most important ginsenosides. Hsieh et al. [42] found that Rh2 can ameliorate LPS-induced lung injury by blocking the TLR4/PI3K/Akt/mTOR, Raf-1/MEK/ERK, and Keap1/Nrf2/HO-1 signaling pathways in mice.

**Figure 3:**
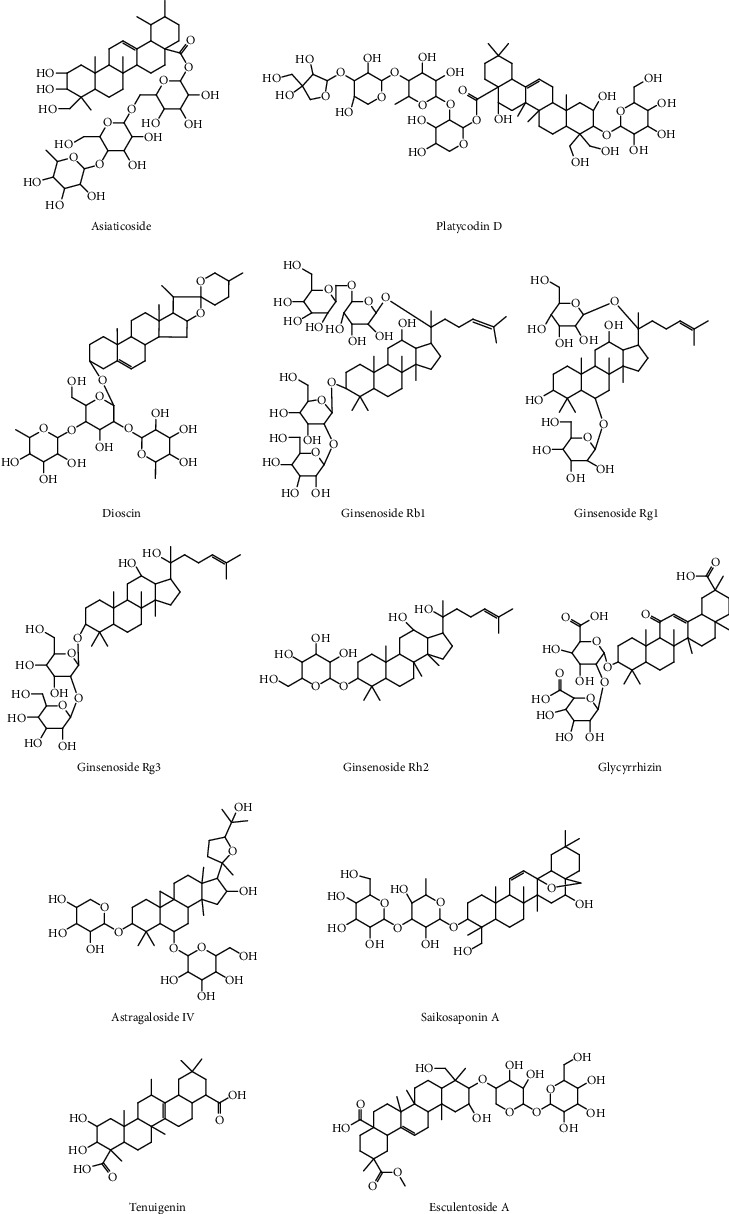
Chemical structures of representative saponins.

Glycyrrhizin is an extractive component isolated from *Glycyrrhiza glabra L*. roots and has various pharmacological effects. It exerts protective effects in various ALI models. In a radiation-induced ALI model, glycyrrhizin mitigated radiation-induced ALI by suppressing the HMGB1/TLR4 pathway [43]. In a LPS-induced ALI model, glycyrrhizin exerted anti-inflammatory effects by suppressing TLR4/NF-*κ*B signaling [44]. In a *Streptococcus aureus*-induced ALI model, glycyrrhizin mitigated lung inflammation after *Streptococcus aureus* infection by inhibiting NF-*κ*B, p38/ERK pathways, and pyroptosis [45].  Table 3 shows the potential effects of saponins from herbs on ALI. The structures of representative saponins are shown in Figure 3. By summarizing the structure-activity relationship of saponins, the anti-inflammatory activities of the compounds were found to be closely related to the types of substituted sugar groups and to increase with the number of substituted sugar groups. In addition, the positions and number of hydroxyl groups on the mother nucleus have a strong influence on the anti-inflammatory activities of saponins [46].

### 2.4. Alkaloids

Alkaloids are nitrogen-containing organic compounds that exist widely in nature. Most alkaloids have special and significant physiological activities, such as anti-inflammatory, antiviral, antitumor, and immune regulation activities [47, 48].

Berberine, an isoquinoline alkaloid, is extracted from *Coptis chinensis* and other Berberis plants. It can reduce lung histopathological changes through the protein kinase R-like endoplasmic reticulum kinase-mediated Nrf2/HO-1 signaling axis [49]. Berberine pretreatment can diminish CS-mediated lung inflammation by inhibiting the secretion of proinflammatory factors and NF-*κ*B activity [50].

Sinomenine is an isoquinoline alkaloid extracted from *Sinomenium acutum*. It can provide protection against *E. coli*-induced ALI in mice by activating Nrf2 and inhibiting NF-*κ*B pathways [51] and can effectively attenuate severe septic-associated ALI possibly by suppressing inflammation and oxidative damage through the activation of autophagy and Nrf2 signaling [52].

Matrine is the main active ingredient of *Sophora flavescens*. Liou et al. [53] found that it can prevent ALI in LPS-induced mice by decreasing the expression levels of COX-2, intercellular cell adhesion molecule-1 (ICAM-1), TNF-*α*, and IL-6.

Protostemonine is an active alkaloid mainly extracted from *Stemona sessilifolia*. In mice treated with protostemonine, heat-killed methicillin-resistant *S. aureus-* (HKMRSA-) mediated ALI can be alleviated through the suppression of the MAPK and NF-*κ*B pathways [54]. Protostemonine can also effectively ameliorate LPS-induced lung inflammatory responses; the beneficial effects may be related to the downregulation of MAPK and AKT phosphorylation and reduction in the expression levels of proinflammatory mediators (such as NO, iNOS, and cytokines) [55].

**Figure 4:**
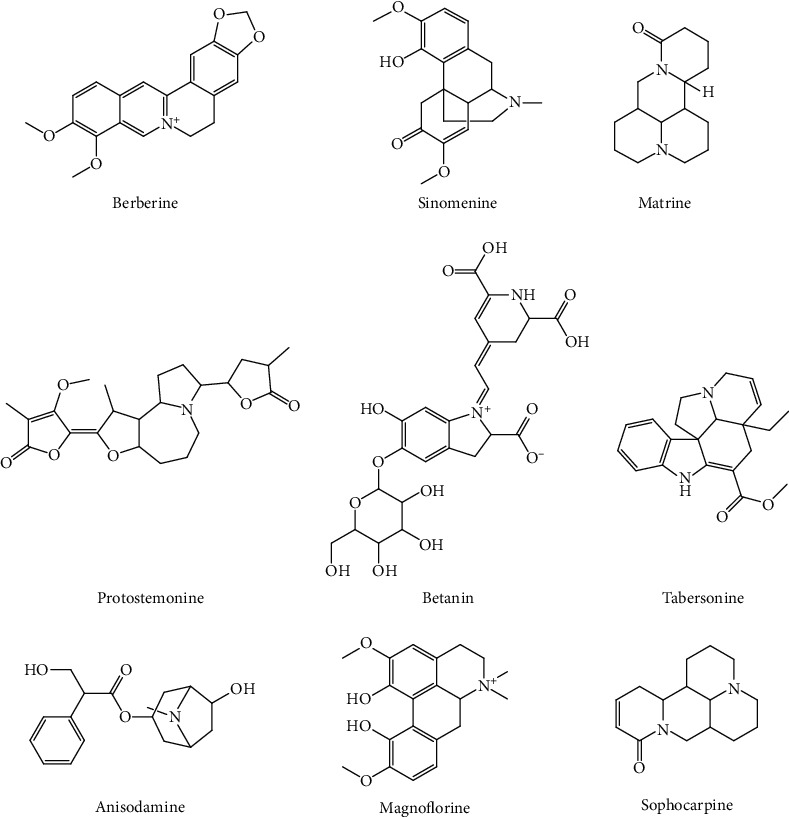
Chemical structures of representative alkaloids.

Betanin is a quaternary ammonium alkaloid, mainly present in red beet roots. Treatment with betanin can protect rats from PQ-induced ALI interstitial pneumonia. The mechanisms may be associated with the increased levels of zonula occluden-1 and claudin-4, decreased levels of TNF-*α* and IL-1, and inhibited NF-*κ*B activity [56].  Table 4 shows the potential effects of alkaloids from herbs on ALI. The structures of representative alkaloids are shown in Figure 4. The structural factors affecting the antioxidant activity of alkaloids are mainly stereostructure and electrical effects. The “exposure” of the nitrogen atoms in the heterocyclic ring facilitates reactions with ROS, resulting in antioxidant effects. Electron-donating groups in alkaloids or structural factors that can enrich nitrogen atoms with electrons can increase antioxidant activity [57]. Borcsa et al. [58] found that the chain length and unsaturation of the C-8 ester groups of aconitine derivatives play important roles in the anti-inflammatory activity. The toxicity of its derivative with a substituted long-chain ester group at the C-8 position is reduced relative to that of aconitine.

### 2.5. Quinonoids

Quinonoids form a large class of natural bioactive molecules with unsaturated cyclodiketone structures (quinone structures) and are mainly divided into four types: benzoquinones, phenanthrenequinones, naphthoquinones, and anthraquinones. Quinonoids exhibit multiple pharmacological activities, such as antibacterial, anti-inflammatory, antiviral, and anticancer effects, and especially play a crucial role in ALI prevention and treatment [59, 60].

Emodin is an anthraquinone derivative isolated from rhubarb and has protective effects in various lung injury models. Dong et al. [61] found that emodin can reactivate autophagy and alleviate inflammatory lung injury in mice with lethal endotoxemia. Another study showed that emodin might provide protection against severe acute pancreatitis- (SAP-) associated ALI by reducing the expression of pre-B-cell colony-enhancing factor and enhancing neutrophil apoptosis through mitochondrial and death receptor apoptotic pathways [62]. Meanwhile, emodin exhibits protective effects on CLP-induced ALI in rats, and the molecular mechanism may be associated with the suppression of p38 MAPK signaling and reduction in oxidative damage and inflammation responses [63]. Moreover, emodin can also attenuate CS-mediated oxidative damage and lung inflammation in mouse models by inhibiting ROS production [64].

Chrysophanol is one of the main active ingredients of rhubarb. Treatment with chrysophanol can attenuate PQ-induced lung inflammation by increasing PPAR-*γ* expression and blocking the NF-*κ*B pathway [65].

Tanshinone IIA is a derivative of phenanthrenequinone isolated from the root of *Salvia miltiorrhiza*. It can prevent LPS-induced expression and proinflammatory mediator secretion, thus exerting a protective effect against ALI probably through the regulation of the Sirt1/NF-*κ*B pathway [66]. Wang et al. [67] investigated the effect and mechanism of tanshinone IIA on PQ-induced ALI. The results indicated that tanshinone IIA exerted a therapeutic effect on ALI rats by decreasing the levels of Ang-(1-7) and angiotensin-converting enzyme 2 (ACE2) in the lungs. Tanshinone IIA also exerts a marked protective effect on seawater aspiration-mediated ALI partly through the downregulation of macrophage migration inhibitory factor and NF-*κ*B activity and TNF-*α* and IL-6 expression [68].

Cryptotanshinone is one of the major active compounds of *Salvia miltiorrhiza*. Cryptotanshinone pretreatment can significantly inhibit LPS-induced neutrophil infiltration in lung tissues. The anti-inflammatory effects of cryptotanshinone may be due to its ability to suppress TLR4-mediated NF-*κ*B signaling [69]. Moreover, cryptotanshinone treatment can ameliorate radiation-induced ALI by suppressing the production and secretion of inflammatory cytokines and inhibiting the activation of CC chemokine ligand 3 (CCL3)/CC chemokine receptor 1 (CCR1) [70].

Embelin is a naturally occurring hydroxybenzoquinone mainly obtained from *Embelia ribes*. Embelin treatment can efficiently relieve PQ-incited lung damage, and the mechanism may be related to the inhibition of oxidative stress, inflammation cascade, and MAPK/NF-*κ*B signaling pathway [71].

**Figure 5:**
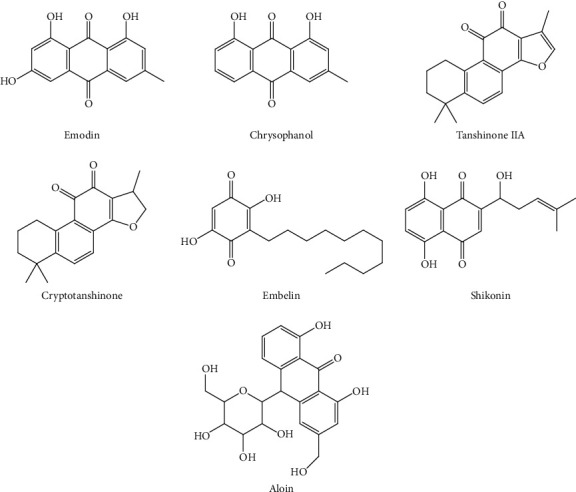
Chemical structures of representative quinonoids.

Shikonin is a naphthoquinone isolated from *Lithospermum*. Treatment with shikonin can improve sepsis-induced ALI by increasing miRNA-140-5p expression, depressing TLR4 expression, and suppressing the expression of downstream MyD88 and NF-*κ*B [72]. Myeloid differentiation protein 2 (MD2) plays a key role in mediating inflammation. Zhang et al. [73] found that shikonin can inhibit the formation of the MD2-TLR4 complex in an ALI mouse model, thereby reducing LPS-mediated inflammation.  Table 5 shows the potential effects of quinonoids from herbs on ALI. The structures of representative quinonoids are shown in Figure 5. Structure-activity relationship analysis showed that the number and locations of OH groups on the benzene rings of quinonoids seem to be largely responsible for the observed large variations in the trends of antioxidative and anti-inflammatory properties. Nam et al. [74] found that compared with quinonoids without OH or only two OH groups on their benzene rings, purpurin with three OH groups, two of which are located in the ortho position of the anthraquinone molecule, exhibited the highest activity in antioxidative and anti-inflammatory cell assays. In addition, glycosides display lower activities than their aglycons [75].

### 2.6. Other Components

**Figure 6:**
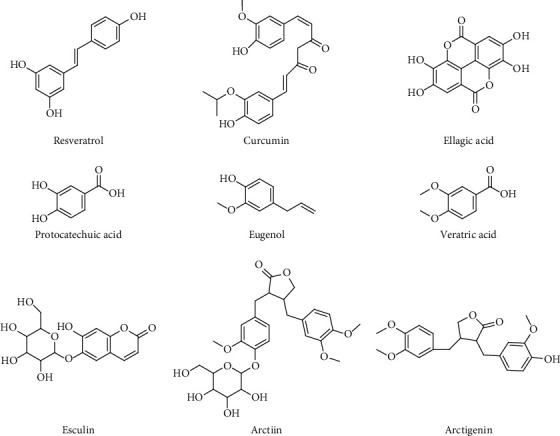
Chemical structures of other active ingredients.

In addition to flavonoids, saponins, terpenoids, alkaloids, and quinonoids, other active ingredients, such as phenols, organic acids, coumarins, and lignans, also play important roles in ALI prevention and treatment (Table 6). The structures of other representative components are shown in Figure 6.

Resveratrol is a stilbene derivative mainly present in *Vitis viniferae fructus*. It exhibits protective effects in various ALI models. In a LPS-mediated ALI model, resveratrol can counteract lung inflammation by suppressing CD45^+^ Siglec F^−^ and CD45^+^ CD206^−^ M1 subtype macrophages [76]. In a sepsis-induced ALI model, resveratrol can suppress the production of proinflammatory cytokines and the apoptosis of alveolar macrophages through the activation of the vascular endothelial growth factor-B (VEGF-B) pathway, thereby exhibiting anti-inflammatory and antiapoptotic effects [77]. In a staphylococcal enterotoxin B- (SEB-) induced ALI model, resveratrol can ameliorate ALI and decrease mortality through miR-193a regulation that targets the TGF-*β* pathway [78].

Curcumin is a natural polyphenolic compound found in *Curcuma longa*. Curcumin pretreatment can relieve the severity of ALI and uncontrolled inflammation in CLP-induced septic mice by enhancing the differentiation of naïve CD4^+^ T cells into CD4^+^ CD25^+^ FOXP3^+^ Treg cells [79]. Moreover, curcumin can significantly improve BLM-induced ALI by downregulating the levels of epidermal growth factor receptor (EGFR) and Ki 67 both in vitro and in vivo [80].

Ellagic acid, a naturally occurring polyphenol, is present in a variety of plants such as eucalyptus, green tea, and geranium. In preventive and therapeutic treatments, ellagic acid can restrain the development of ALI induced by HCl by reducing IL-6 levels and increasing IL-10 levels in BALF [81].

Protocatechuic acid, a dihydroxybenzoic acid, is a phenolic acid found in plants such as *Alpinia oxyphylla*. It can effectively ameliorate lung histopathological changes induced by LPS by suppressing the p38 MAPK and NF-*κ*B signaling pathways [82].

Eugenol is a natural phenolic substance found in medicinal plants, such as cinnamon, clove, and bay leaves. Eugenol can alleviate LPS-induced ALI by suppressing the release of inflammatory mediators (IL-1*β*, TNF-*α*, and IL-6), NADPH oxidase activity, and antioxidant enzyme activity [83].

Veratric acid is a benzoic acid extracted from *Trollius chinensis Bunge*. It can attenuate inflammatory injury caused by LPS by inhibiting NF-*κ*B signaling pathways [84].

Esculin, a coumarin compound isolated from *Fraxini cortex*, can inhibit LPS-induced lung inflammation in mice by regulating the TLRs/MyD88/NF-*κ*B signaling pathways [85].

Arctiin and arctigenin are lignan compounds isolated from *Arctium lappa*. Arctiin can prevent LPS-induced ALI in mice by inhibiting the PI3K/AKT/NF-*κ*B signaling pathway [86], and arctigenin can provide protection against LPS-induced lung inflammation and oxidative stress in a mouse model by suppressing the MAPK, iNOS, and HO-1 pathway [87].

## 3. Discussion

By organizing the active ingredients from herbs and their roles in preventing and treating ALI, the mechanisms can be summarized as follows:

### 3.1. Improvement of Pathological Changes

#### 3.1.1. Inhibition of the Inflammatory Response

Under ALI, neutrophils, macrophages, vascular endothelial cells, and many other immune cells are activated in the body to release a series of inflammatory mediators, leading to an uncontrolled systemic inflammatory response [1]. This activation is one of the important mechanisms of ALI prevention and control with herbal active ingredients, regulation of the levels of inflammatory mediators, and balancing of pro- and anti-inflammatory responses [70, 81].

#### 3.1.2. Amelioration of the Barrier Function

The main pathological features of ALI include pulmonary edema caused by the destruction of the alveolar-capillary barrier and imbalance in the inflammatory response caused by leukocyte recruitment [2]. The destruction of alveolar epithelial cells increases barrier permeability and decreases the clearance rate of alveolar fluid; injury to vascular endothelial cells leads to fluid and macromolecules entering the gap, thus causing pulmonary edema. Herbal active ingredients, such as platycodin D and astragaloside IV, can maintain the integrity of the barrier function by inhibiting the apoptosis of alveolar epithelium or endothelial cells [32, 88].

#### 3.1.3. Alleviation of Oxidative Stress

Under ALI, activated neutrophils release a large amount of ROS. The release of considerable ROS, on the one hand, directly damages the unsaturated fatty acids in the cell membrane, reduces the fluidity of the membrane, and increases permeability. On the other hand, it can release substantial oxygen-free radicals to lung tissue, which directly damages alveolar epithelial and pulmonary vascular endothelial cells, destroys the blood barrier, and aggravates pulmonary edema [89]. Therefore, reconstruction of the balance between oxidation and antioxidation can ease ALI. Studies have shown that herbal active ingredients, including tectorigenin and arctigenin, can ease oxidative stress and pathological damage to the lung by increasing the contents of SOD and HO-1 [7, 87].

### 3.2. Regulation of ALI-Related Pathways

#### 3.2.1. Inhibition of the NF-*κ*B Pathway

When the ALI occurs, the body will produce a large number of inflammatory factors. Their transcription is mainly related to NF-*κ*B, a key nuclear transcription factor. In resting cells, NF-*κ*B and I*κ*B form a compound body that exists in the cytoplasm in an inactive form. When the cell is stimulated by extracellular signals, I*κ*B phosphorylates and free NF-*κ*B rapidly shift to the nucleus, thereby inducing the transcription of related genes, including proinflammatory factors IL-6 and TNF-*α*, and aggravating ALI [90]. Many researches demonstrated that some herbal active ingredients can improve ALI by inhibiting the NF-*κ*B pathway [29, 50, 84].

#### 3.2.2. Inhibition of the MAPK Pathway

MAPK activation is crucial in the occurrence and development of ALI. MAPK includes three main subfamilies, namely, ERK, JNK, and p38, which can regulate cytokine release and signal transduction in ALI. JNK and p38 can regulate the occurrence of proinflammatory factors IL-6 and TNF-*α*. p38 can regulate the activation of NF-*κ*B induced by LPS by inhibiting neutrophil migration and aggravate the disease condition of ALI patients [91]. Hesperidin, emodin, and other herbal active ingredients can alleviate the development of ALI by inhibiting the MAPK pathway [12, 63].

#### 3.2.3. Other Pathway

**Figure 7:**
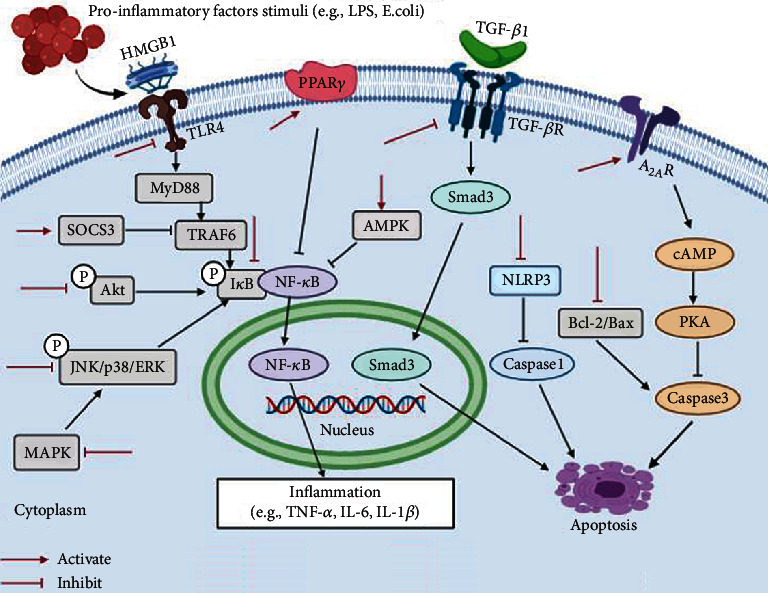
Schematic presentation of the anti-ALI mechanisms of herbal active ingredients.

Herbal active ingredients can also affect the occurrence and development of ALI by regulating Akt, AMPK, cAMP/PKA, or TGF-*β* pathways [16, 40, 78, 92]. The anti-ALI mechanisms of herbal active ingredients are summarized in Figure 7.

### 3.3. Regulation of ALI-Related Receptors

#### 3.3.1. Inhibition of TLRs

TLRs are important proteins involved in nonspecific immunity. TLR2 and TLR4 can recognize LPS and play an important role in LPS-mediated inflammatory responses. After invading the lung, LPS binds to the TLR2 or TLR4 receptor, which activates the NF-*κ*B pathway and induces inflammation. Therefore, inhibition of TLR2 or TLR4 activation can ease immune and inflammatory responses [93]. Studies have shown that hydroxysafflor yellow A and ginsenoside Rb1 can alleviate ALI by inhibiting the expression of TLR4 or TLR2 [37, 94].

#### 3.3.2. Upregulation of PPAR-*γ*

The imbalance in inflammatory response and immune regulation can promote the occurrence and development of ALI. Studies have revealed that activated PPAR-*γ* has anti-inflammatory and immunoregulation effects [95, 96]. Triptolide and citral can inhibit the activation of NF-*κ*B by increasing the content of PPAR-*γ*, thus improving the inflammatory response of ALI [97, 98].

#### 3.3.3. Activation of the Nuclear Receptor LXR*α*

LXR*α*, one of the main members of the nuclear receptor superfamily, can selectively reduce the expression of pulmonary inflammatory factors when activated and inhibit the activity of neutrophils and macrophages, which play a key role in the ALI inflammatory response. It can also increase the expression of several immune proteins and antioxidant enzymes in the lung. Therefore, LXR*α* is a potential anti-ALI target [99]. Researches have shown that acanthoic acid and platycodin D can alleviate lung inflammation by activating LXR*α* [31, 100].

## 4. Conclusion and Outlook

Herbal active ingredients for preventing and treating ALI mainly include flavonoids, terpenoids, saponins, alkaloids, and quinonoids, and animal models are used to verify their efficacy. The LPS-induced ALI model is the most widely used, and the main modeling methods include intraperitoneal injection, tracheal instillation, nasal instillation, and tail intravenous injection. The cecal ligation and puncture-induced in vivo sepsis model, retrograde pancreaticobiliary duct injection of taurocholic acid-induced severe acute pancreatitis model, tail intravenous injection of oleic acid, tracheal instillation of BLM, inhalation of influenza virus, and intraperitoneal injection of PQ are also to induce the ALI model. Although the verification of activity is simple at the cellular level, multicell interaction during ALI occurrence cannot be stimulated accurately. Most laboratories use Western blot and other methods to investigate changes in the protein contents of related pathways, but interference or knockout is rarely used in confirming the specificity of proteins and their related upstream and downstream proteins. The inhibitors of related pathways are also seldom used. Therefore, clarifying the possible targets of the active ingredients from herbs seems difficult.

In summary, herbal active ingredients possess anti-ALI activity and can ameliorate ALI by regulating immune cell function, inhibiting inflammatory response, improving barrier function, and modulating related signal transduction pathways. Further studies are needed to confirm the action target and possible structural modification and to provide insights and references for the discovery of novel drugs for ALI prevention and treatment.

## Figures and Tables

**Table 1:** Potential effects of flavonoids on ALI.

Active ingredients	Models of ALI	Doses (mg/kg)	Relevant findings	Ref.
Naringin	ALI in mice induced by PQ	60 and 120	Reduce the levels of TNF-*α*, TGF-*β*1, TIMP-1, and MMP-9	[5]
	ALI in mice induced by LPS ALI in dogs induced by LPS	15 and 60 12.4	Reduce airway mucus secretion and goblet cell hyperplasia, promote sputum excretion	[6]
Tectorigenin	ALI in mice induced by LPS	5 and 10	Decrease the expression of NF-*κ*B p65 at both mRNA and protein levels, improve SOD activity, and inhibit MPO activity	[7]
Baicalin	ALI in mice induced by LPS	200	Activate Nrf2/HO-1 pathway	[8]
	ALI in rats induced by severe burn	80	Modulate NLRP3 inflammasome pathways	[9]
	ALI in chicken induced by APEC	50, 100, and 200	Inhibit NF-*κ*B pathway	[10]
Hesperidin	ALI in mice induced by CLP	10 and 20	Induce the Hsp70/TLR4/MyD88 pathway	[11]
	ALI in mice induced by H1N1	50, 200, and 500	Reduce proinflammatory cytokine production by inhibiting MAPK pathways	[12]
	ALI in mice induced by LPS	500	Suppress HMGB1 expression and release	[13]
Apigenin	ALI in mice induced by PQ	20 and 50	Increase the level of MDA and decrease the activity of antioxidase	[14]
	ALI in mice induced by LPS	10 and 20	Inhibit the activation of COX-2 and NF-*κ*B	[15]
Rutin	ALI in mice induced by LPS	0.61, 6.1, and 61	Suppress the expressions of VCAM-1 and iNOS	[101]
Troxerutin	ALI in mice induced by LPS	150	Inhibit MAPK and NF-*κ*B pathways	[102]
Hydroxysafflor yellow A	ALI in rats induced by OA	15	Activate cAMP/PKA pathway	[103]
	ALI in mice induced by LPS	40, 80, and 120	Inhibit TLR4-dependent pathways	[92]
	ALI in mice induced by BLM	26.7, 40, and 60	Inhibit NF-*κ*B activation and p38 MAPK phosphorylation	[94]
EGCG	ALI in mice induced by LPS	10	Suppress TLR4/NF-*κ*B pathway	[104]
	ALI in mice induced by PQ	5, 10, and 20	Inhibit TLR/NF-*κ*B pathway	[105]
	ALI in mice induced by H9N2	10	Inhibit TLR4/NF-*κ*B pathway	[106]
Kaempferol	ALI in mice induced by LPS	50	Modulate TRAF6 polyubiquitination	[107]
	ALI in mice induced by CLP	100	Suppress oxidative stress, iNOS, and ICAM-1 pathways	[108]
	ALI in mice induced by H9N2	15	Inhibit TLR4/MyD88/NF-*κ*B and MAPK pathways	[109]
Luteolin	ALI in mice induced by LPS	1 and 10	Inhibit the activity of iNOS/NO, COX-2, HMGB1, and NF-*κ*B	[110]
	ALI in mice induced by HgCl_2_	100	Prevent NF-*κ*B activation and activate AKT/Nrf2 pathway	[111]
Quercetin	ALI in mice induced by LPS	25 and 50	Activate cAMP-Epac pathway	[112]
	ALI in rats induced by CLP	30 and 50	Inhibit ICAM-1 and MIP-2 expression	[113]
	ALI in mice induced by seawater instillation	200	Inhibit macrophage M1 polarization and proinflammatory cytokine expression	[114]
Dihydromyricetin	ALI in mice induced by CLP	50, 100, and 150	Inhibit NLRP3 inflammasome-dependent pyroptosis	[115]
Isoliquiritigenin	ALI in mice induced by LPS	30	Activate AMPK/Nrf2/ARE signaling and inhibit NF-*κ*B and NLRP3 activation	[116]

**Table 2:** Potential effects of terpenoids on ALI.

Active ingredients	Models of ALI	Doses (mg/kg)	Relevant findings	Ref.
Andrographolide	ALI in mice induced by OVA	5 and 10	Suppress ROS-mediated NF-*κ*B signaling and NLRP3 inflammasome activation	[19]
	ALI in mice induced by radiation	5, 10, and 20	Suppress AIM2 inflammasome-mediated pyroptosis in macrophage	[20]
	ALI in mice induced by CS	0.1, 0.5, and 1	Augment the activity of Nrf2	[21]
Oridonin	ALI in mice induced by LPS	20 and 40	Exert protective effects through Nrf2-independent anti-inflammatory and Nrf2-dependent antioxidative activities	[22]
	ALI in mice induced by hyperoxia	10	Reduce MDA and TNF-*α*, and increase GSH and IL-10 in the lungs	[23]
Betulin	ALI in rats induced by CLP	4 and 8	Inhibit NF-*κ*B and MAPK pathway	[24]
Betulinic acid	ALI in mice induced by CLP	3, 10, and 30	Decrease the levels of oxidants, increase the levels of antioxidants in the lungs and plasma	[25]
Asiatic acid	ALI in mice induced by LPS	25, 50, and 100	Block the TLR4/NF-*κ*B pathway	[26]
	ALI in rat induced by SCI	30 and 75	Inhibit NLRP3 inflammasome activation and oxidative stress with the upregulation of Nrf2	[117]
Acanthoic acid	ALI in mice induced by LPS	15, 30, and 60	Upregulate the expression of LXR*α*	[100]
Paclitaxel	ALI in mice induced by CLP	0.075, 0.150, 0.225, and 0.300	Activate MUC1 and suppress TLR4/NF-*κ*B pathway	[118]
Geraniol	ALI in mice induced by LPS	12.5, 25, and 50	Inhibit TLR4-mediated NF-*κ*B and Bcl-2/Bax pathways	[119]
Triptolide	ALI in mice induced by LPS	0.005, 0.010, and 0.015	Activate PPAR-*γ*	[97]
Citral	ALI in mice induced by LPS	10, 20, and 40	Activate PPAR-*γ*	[98]
Geniposide	ALI in mice induced by LPS	20, 40, and 80	Block NF- *κ*B and MAPK pathway	[120]
Jolkinolide B	ALI in mice induced by LPS	2 and 10	Suppress the activation of NF-*κ*B and MAPK	[121]
Taraxasterol	ALI in mice induced by LPS	2.5, 5, and 10	Inhibit the NF-*κ*B and MAPK pathways	[122]

**Table 3:** Potential effects of saponins on ALI.

Active ingredients	Models of ALI	Doses (mg/kg)	Relevant findings	Ref.
Asiaticoside	ALI in mice induced by LPS	15, 30, and 45	Inhibit NF-*κ*B pathway	[29]
	ALI in mice induced by CLP	45	Upregulate PPAR-*γ* expression, inhibit MAPK and NF-*κ*B pathway	[30]
Platycodin D	ALI in mice induced by LPS	20, 40, and 80	Activate LXR*α*-ABCA1 pathway	[31]
	ALI in mice induced by LPS or BLM	50 and 100	Suppress apoptosis and inflammation	[32]
Dioscin	ALI in mice induced by BLM	80	Attenuate oxidative stress and the inflammatory response	[123]
	ALI in mice induced by LPS	20, 40, and 80	Inhibit NF-*κ*B activation as well as TLR4 expression	[33]
Ginsenoside Rb1	ALI in mice induced by *S. aureus*	10 and 20	Attenuate NF-*κ*B and MAPK activation	[37]
Ginsenoside Rg1	ALI in mice induced by CLP	10 and 20	Upregulate SIRT1 to relieve ER stress and inflammation	[38]
	ALI in rats induced by IR	40	Regulate NF-*κ*B/COX-2 pathway	[39]
Ginsenoside Rg3	ALI in mice induced by LPS	10, 20, and 30	Activate MerTK-dependent PI3K/AKT/mTOR pathway	[40]
	ALI in rats induced by omethoate	5, 10, and 20	Reduce inflammation and oxidation	[41]
Ginsenoside Rh2	ALI in mice induced by LPS	5, 10, and 20	Regulate the TLR4/PI3K/Akt/mTOR, Raf-1/MEK/ERK, and Keap1/Nrf2/HO-1 pathways	[42]
Glycyrrhizin	ALI in mice induced by radiation	10	Inhibit the HMGB1/TLR4 pathway	[43]
	ALI in mice induced by LPS	50	Inhibit the TLR4/NF-*κ*B pathway	[44]
	ALI in mice induced by *S. aureus*	25	Inhibit NF-*κ*B, p38/ERK pathways, and pyroptosis	[45]
Astragaloside IV	ALI in rats induced by CLP	2.5, 5, and 10	Improve pulmonary ventilation, decrease alveolar-capillary permeability	[88]
	ALI in mice induced by PQ	50 and 100	Suppress Rho/ROCK/NF-*κ*B pathway	[124]
Saikosaponin A	ALI in mice induced by LPS	5, 10, and 20	Inhibit NF-*κ*B and NLRP3 pathways	[125]
Tenuigenin	ALI in mice induced by LPS	2, 4, and 8	Inhibit NF-*κ*B and MAPK pathways	[126]
Esculentoside A	ALI in mice induced by LPS	15, 30, and 60	Inhibit NF-*κ*B and MAPK pathways	[127]

**Table 4:** Potential effects of alkaloids on ALI.

Active ingredients	Models of ALI	Doses (mg/kg)	Relevant findings	Ref.
Berberine	ALI in mice induced by LPS	10	Activate PERK-mediated Nrf2/HO-1 signaling axis	[49]
	ALI in mice induced by CS	50	Inhibit NF-*κ*B activation	[50]
Sinomenine	ALI in mice induced by *E. coli*	100	Inhibit the activation of NF-*κ*B and upregulate the expression of Nrf2	[51]
	ALI in mice induced by LPS	100	Activate Nrf2 and autophagy	[52]
Matrine	ALI in mice induced by LPS	10 and 20	Decrease the expressions of COX-2 and ICAM-1	[53]
Protostemonine	ALI in mice induced by HKMRSA	20	Inhibit MAPK and NF-*κ*B pathways	[54]
	ALI in mice induced by LPS	10	Inhibit iNOS and NO expression, and suppress MAPK and PI3K/AKT signaling transduction in macrophages	[55]
Betanin	ALI in rats induced by PQ	25 and 100	Decrease the levels of IL-1 and TNF-*α*, and the activity of NF-*κ*B	[56]
Tabersonine	ALI in mice induced by LPS	20 and 40	Reduce the K63-linked polyubiquitination of TRAF6	[128]
Anisodamine	ALI in rats induced by LPS	10	Inhibit the levels of IL-17A and IL-17F	[129]
Magnoflorine	ALI in mice induced by LPS	5, 10, and 20	Suppress TLR4-mediated NF-*κ*B and MAPK activation	[130]
Sophocarpine	ALI in mice induced by LPS	12.5, 25, and 50	Inhibit TLR4-mediated NF-*κ*B and MAPK pathways	[131]

**Table 5:** Potential effects of quinonoids on ALI.

Active ingredients	Models of ALI	Doses (mg/kg)	Relevant findings	Ref.
Emodin	ALI in mice induced by LPS	20	Upregulate the expression of BECN1 and LC3-II	[61]
	ALI in rats induced by SAP	10	Decrease the expression of PBEF	[62]
	ALI in rats induced by CLP	25	Inhibit p38 MAPK pathway, and reduce oxidative stress and inflammation response	[63]
	ALI in mice induced by CS	20 and 40	Enhance the expressions and activities of HO-1 and Nrf-2	[64]
Chrysophanol	ALI in mice induced by PQ	10 and 20	Activate PPAR-*γ* and inhibit NF-*κ*B pathway	[65]
Tanshinone IIA	ALI in mice induced by LPS	10	Modulate Sirt1/NF-*κ*B pathway	[66]
	ALI in rats induced by PQ	25	Enhance the levels of ACE2 and Ang-(1-7)	[67]
	ALI in rats induced by seawater aspiration	10	Downregulate MIF and the activity of NF-*κ*B	[68]
Cryptotanshinone	ALI in mice induced by LPS	10, 20, and 40	Inhibit TLR4-mediated NF-*κ*B pathways	[69]
	ALI in rats induced by radiation	20	Regulate the production and release of inflammatory cytokines especially MMP-1, and inhibit the activation of CCL3/CCR1	[70]
Embelin	ALI in rats induced by PQ	20	Suppress oxidative stress and inflammatory cascade by modulating MAPK/NF-*κ*B pathway	[71]
Shikonin	ALI in rats induced by LPS	12.5, 25, and 50	Regulate miRNA-140-5p/TLR4-a	[72]
	ALI in mice induced by LPS	12.5 and 25	Inhibit MD2-TLR4 complex formation	[73]
Aloin	ALI in mice induced by LPS	1.6~12.4	Reduce inflammatory gene iNOS by inhibition activity and p-STAT-1 and NF-*κ*B	[132]

**Table 6:** Potential effects of other components on ALI.

Active ingredients	Models of ALI	Doses (mg/kg)	Relevant findings	Ref.
Resveratrol	ALI in murine induced by LPS	30	Activate SOCS3 pathway	[76]
	ALI in mice induced by CLP	40	Activate the VEGF-B pathway	[77]
	ALI in mice induced by SEB	100	Downregulate miR-193a that targets TGF-*β* pathway	[78]
Curcumin	ALI in mice induced by CLP	20	Enhance Treg cell differentiation and increase IL-10 production	[79]
	ALI in mice induced by BLM	75	Downregulate the expression of Ki 67 and EGFR	[80]
Ellagic acid	ALI in mice induced by HCl	10	Downregulate the level of IL-6 and upregulate the level of IL-10	[81]
Protocatechuic acid	ALI in mice induced by LPS	5, 15, and 30	Suppress p38MAPK and NF-*κ*B pathways	[82]
Eugenol	ALI in mice induced by LPS	150	Suppress the release of inflammatory factors and the activity of antioxidant enzymes	[83]
Veratric acid	ALI in mice induced by LPS	12.5, 25, and 50	Inhibit NF-*κ*B pathway	[84]
Esculin	ALI in mice induced by LPS	20 and 40	Inhibit TLR/NF-*κ*B pathway	[85]
Arctiin	ALI in mice induced by LPS	10, 20, and 40	Suppress PI3K/AKT/NF-*κ*B pathway	[86]
Arctigenin	ALI in mice induced by LPS	50	Suppress MAPK, HO-1, and iNOS pathway	[87]

## Abbreviations

ALI: Acute lung injury
ABCA1: ATP-binding cassette transporter A1
AIM2: Absent in melanoma 2
ACE2: Angiotensin-converting enzyme 2
BALF: Bronchoalveolar lavage fluids
Bcl-2: B-cell lymphoma-2
Bax: Bcl2-associated X protein
BLM: Bleomycin
CAT: Catalase
COX-2: Cyclo-oxygen-ase-2
CLP: Cecal ligation and puncture
CS: Cigarette smoke
CCL3: CC chemokine ligand 3
CCR1: CC chemokine receptor 1
E. coli: Escherichia coli
EGCG: Epigallocatechin-3-gallate
ERK: Extracellular regulated kinases
ER: Endoplasmic reticulum
EGFR: Epidermal growth factor receptor
GSH-Px: Glutathione peroxidase
HKMRSA: Heat-killed methicillin-resistant Staphylococcus aureus
Hsp70: Heat shock protein 70
HO-1: Heme oxygenase-1
HMGB1: High mobility group box-1 protein
IR: Ischemia-reperfusion
IL: Interleukin
ICAM-1: Intercellular cell adhesion molecule-1
JNK: c-Jun N-terminal kinase
LPS: Lipopolysaccharides
LXR*α*: Liver x receptor *α*
MD2: Myeloid differentiation protein 2
MMP-9: Matrix metalloprotein-9
MPO: Myeloperoxidase
MyD88: Myeloid differential protein-88
MAPK: Mitogen-activated protein kinase
MDA: Malondialdehyde
NF-*κ*B: Nuclear factor kappa b
Nrf2: Nuclear factor E2-related factor 2
NLRP3: Nod-like receptor pyrin domain-containing protein 3
OA: Oleic acid
OVA: Ovalbumin
PQ: Paraquat
PPAR-*γ*: Peroxisome proliferator-activated receptors-*γ*
ROS: Reactive oxygen species
SAP: Severe acute pancreatitis
SCI: Spinal cord injury
SEB: Staphylococcal enterotoxin B
SOD: Superoxide dismutase
TGF-*β*: Transforming growth factor-*β*
TNF-*α*: Tumor necrosis factor-*α*
TIMP-1: Tissue inhibitor of metalloproteinase-1
TLR4: Toll-like receptor 4
VEGF-B: Vascular endothelial growth factor-B.
